# A Thermophilic Bacterial Esterase for Scavenging Nerve Agents: A Kinetic, Biophysical and Structural Study

**DOI:** 10.3390/molecules26030657

**Published:** 2021-01-27

**Authors:** Janek Bzdrenga, Elodie Trenet, Fabien Chantegreil, Kevin Bernal, Florian Nachon, Xavier Brazzolotto

**Affiliations:** Département de Toxicologie et Risques Chimiques, Institut de Recherche Biomédicale des Armées, 91220 Brétigny-sur-Orge, France; janek.bzdrenga@def.gouv.fr (J.B.); elodie.trenet@chemdef.fr (E.T.); fabien.chantegreil@def.gouv.fr (F.C.); kevin.bernal@chemdef.fr (K.B.); xavier.brazzolotto@def.gouv.fr (X.B.)

**Keywords:** esterase, thermophilic, X-ray crystallography, nerve agents, bioscavenger

## Abstract

Organophosphorous nerve agents (OPNA) pose an actual and major threat for both military and civilians alike, as an upsurge in their use has been observed in the recent years. Currently available treatments mitigate the effect of the nerve agents, and could be vastly improved by means of scavengers of the nerve agents. Consequently, efforts have been made over the years into investigating enzymes, also known as bioscavengers, which have the potential either to trap or hydrolyze these toxic compounds. We investigated the previously described esterase 2 from *Thermogutta terrifontis* (TtEst2) as a potential bioscavenger of nerve agents. As such, we assessed its potential against G-agents (tabun, sarin, and cyclosarin), VX, as well as the pesticide paraoxon. We report that TtEst2 is a good bioscavenger of paraoxon and G-agents, but is rather slow at scavenging VX. X-ray crystallography studies showed that TtEst2 forms an irreversible complex with the aforementioned agents, and allowed the identification of amino-acids, whose mutagenesis could lead to better scavenging properties for VX. In conjunction with its cheap production and purification processes, as well as a robust structural backbone, further engineering of TtEst2 could lead to a stopgap bioscavenger useful for in corpo scavenging or skin decontamination.

## 1. Introduction

**Figure 1:**
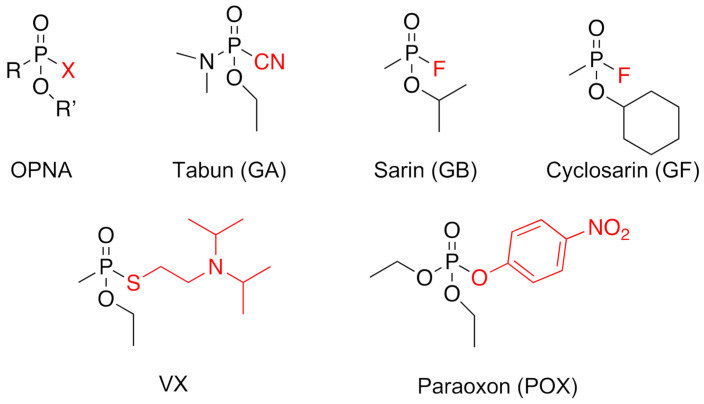
Global chemical structure of organophosphorus nerve agents (OPNA) and specific toxics or pesticides used in the present study. In red is represented the leaving group that is lost upon phosphylation of the enzyme.

Since the Tokyo subway terror attack in 1995, organophosphorus nerve agents (OPNA, Figure 1) [1] such as sarin (GB), VX, or tabun (GA), have been considered as a major risk, not only for soldiers in operations, but also for the civilian population. Despite the ban of these chemical weapons, such a threat has been substantiated as OPNAs have been used, as reported, on several occasions over the past few years: such as during the Syrian conflict, with GB attacks in 2013 and 2017; the VX assassination of the Korean leader’s half-brother in Malaysia in 2017; and more recently with the poisoning of the Skripals in the UK in 2018 and Navalny in Russia in 2020, with a new generation OPNA, called Novichoks. Moreover, organophosphorus compounds are also represented in pesticides and still widely used around the world, causing millions of intoxications per year due to misuse and suicide attempts [2]. These nerve agents specifically target acetylcholinesterase (AChE, 3.1.1.7), a key enzyme situated in the inter-synaptic cleft in the neuro-muscular junction and the central nervous system (CNS) that catalyzes the hydrolysis of the neurotransmitter acetylcholine, thus ensuring an efficient nerve signal transmission. The OPNA reaction leads to the formation of a covalent phosphyl adduct on the catalytic serine, inhibiting the enzyme activity. This results in an accumulation of the neurotransmitter, and a major cholinergic crisis. This physiologically translates to SLUDGEM symptoms (salivation, lacrimation, urination, defecation, gastrointestinal upset, emesis, and miosis) eventually leading to respiratory distress, followed by death, in the absence of intense medical care.

The treatment of OPNA intoxication is a tri-therapy: (a) atropine, an anticholinergic that counteracts the overstimulation of muscarinic receptors; (b) diazepam, an anticonvulsant to protect against the seizures resulting from the cholinergic crisis: and (c) an oxime (2PAM; HI-6, obidoxime). The latter is a strong nucleophile able to displace the phosphyl adduct from the catalytic serine of AChE, thus restoring the enzyme activity. Despite great efforts for many years, the currently used oximes are not efficient on all OPNA-phosphyl adducts, and do not readily cross the blood–brain barrier, thus preventing an efficient action in the CNS. Recent efforts have focused on uncharged [3] or zwitterionic [4] oxime reactivators, in order to favor the CNS tropism of these molecules.

For persistent OPNA such as V agents, for which the percutaneous intoxication route is the most likely, human butyrylcholinesterase (BChE, 3.1.1.8) [5], an enzyme able to rapidly trap the nerve agents before they reach their physiological target, has proven to be an efficient emergency treatment in a guinea-pig animal model [6,7]. The efficient use of BChE as a prophylactic treatment has been also demonstrated in an animal model [8,9]. However, for BChE to act as a stoichiometric bioscavenger, i.e., able to trap only one molecule of toxic per protein molecule, large amounts of protein are necessary to afford an efficient protection. It is estimated that about 200 mg per person would be necessary to protect against 2 LD_50_ of soman [10]. Therefore, efforts have been made to develop large scale recombinant productions [11,12], and to improve the purification process [13,14]. Yet, the cost of a therapeutic dose remains prohibitive for a large diffusion. Bacterial bioscavengers, such as organophosphorus hydrolase (OPH) and its optimized mutants [15,16], are promising candidates, as these enzymes are able to hydrolyze the OPNA at a fast pace, thus theoretically allowing the reduction of the protein amount of the therapeutic dose [17]. However, their bacterial origin implies protein modifications, such as PEGylation, to improve pharmacodynamics and reduce immunogenicity, which mechanically increases the costs of the dose. These enzymes can also be incorporated without modifications into solutions or hydrogels for skin decontamination.

In the present study, we focused on a bacterial esterase as a potential bioscavenger candidate. In order to facilitate the purification costs, we searched the ESTHER database [18] for a thermophilic esterase, and identified the Esterase 2 from *Thermogutta terrifontis* (TtEst2) that has been previously structurally characterized [19], and presents an open active site that could ease protein engineering for further optimizations. The recombinant protein was purified, biochemically characterized, and its kinetic behavior towards different OPNA studied, before performing a structural analysis of its complexes formed when soaked in a racemic solution of the OPNA. These data can shed light on possible modifications of TtEst2, in order to convert it into an efficient bioscavenger or a gentle decontamination product.

## 2. Results

### 2.1. Recombinant Expression and Purification of TtEst2

**Figure 2:**
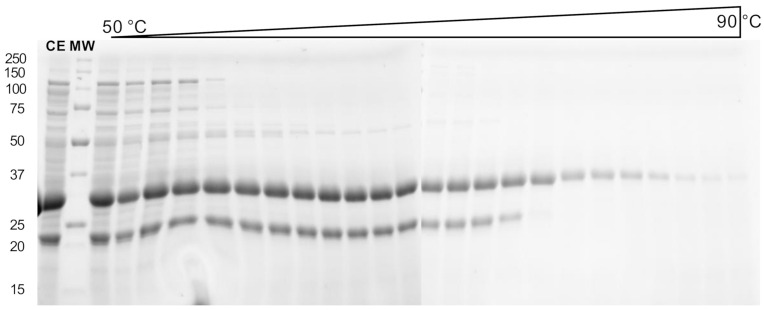
Denaturing condition gel electrophoresis analysis of TtEst2 purification through a heating protocol. CE: crude soluble extract; MW: molecular weight standards (reported in kDa on the left side).

Recombinant expression was carried out using autoinduction growth medium in *Escherichia coli* BL21 (DE3) strain. An attempt to purify TtEst2 by heating the crude extract was carried out. Aliquots of the crude extract were incubated at different temperatures (50–90 °C) for 5 min and centrifugated. Esterase activity was measured in the recovered supernatant. The activity remained stable up to 75 °C, then drastically decreased above 80 °C (data not shown). To correlate the activity evolution to TtEst2 purification, the heated aliquots were analyzed by electrophoresis in denaturing conditions (SDS-PAGE, Figure 2). All aliquots presented an intensive band around 30 kDa, in accordance with the calculated molecular weight of TtEst2 harboring the poly-histidine tag and the TEV cleaving site (33,320 Da), which clearly disappeared around 80 °C. However, the extracts are contaminated with a low molecular weight protein or fragment around 20 kDa, and this contaminant is not easily ridden off until 78 °C, the temperature at which native TtEst2 begins to denature, both in gel and from activity measurements. This narrow temperature window precludes the use of this simple heat protocol to purify TtEst2, without loss or damage to the protein.

**Figure 3:**
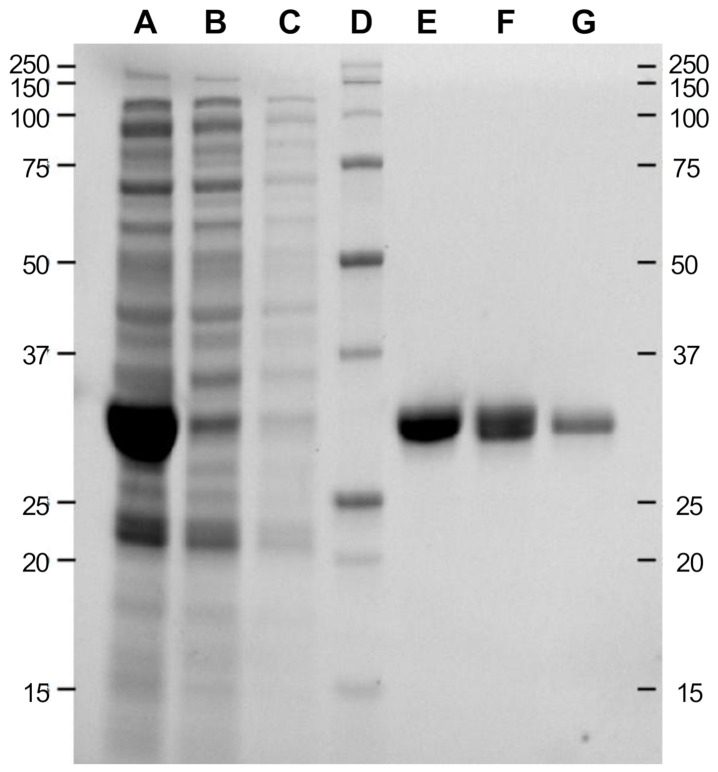
Denaturing condition gel electrophoresis analysis of TtEst2 chromatographic purification fractions. (**A**), crude soluble extract; (**B**), flow-through fraction of the initial metal chelate chromatographic step; (**C**), wash fraction of the initial metal chelate chromatographic step; (**D**), molecular weight standards (weights in kDa are reported on both sides of the gel); (**E**), elution fraction of the initial metal chelate chromatographic step; (**F**), dialysis fraction after TEV protease cleavage; (**G**), flow-through fraction of the second metal chelate chromatographic step.

In order to purify TtEst2 and to get rid of the major low-molecular weight contaminant without losing too much native TtEst2, we submitted the crude extract to two straightforward chromatographic steps, as described in Materials and Methods. Different aliquots were collected throughout the purification process and analyzed by electrophoresis in denaturing conditions (Figure 3). The initial metal-chelate chromatographic step allowed isolation of an already highly pure fraction (Figure 3, lane E). Overnight dialysis was achieved in the presence of recombinant TEV protease, in order to remove the poly-histidine tag and reduce the imidazole concentration for the second chromatographic step. It is hardly necessary to note that this digestion step was incomplete, with the presence of two bands in the dialysis fraction (Figure 3, lane F), and characterized by a recovery of only 44% of the initial esterase activity after the second chromatographic step (Figure 3, lane G). However, despite this unoptimized protocol, TtEst2 was obtained with a yield of 63 mg per liter of autoinduction medium.

### 2.2. Enzymatic Characterization

Enzymatic characterization of TtEst2 was carried out using paranitrophenylpropionate (pNP-C3) as the substrate. pNP-C3 hydrolysis by TtEst2 obeys a classical Michaelis–Menten model, with K_M_ = 1.75 ± 0.24 mM and k_cat_ = 33.9 ± 4.8 s^−1^ (data not shown). This particularly slow hydrolysis implies that concentrations of enzyme in the 0.1 µM range are necessary to perform activity measurements. It follows that inhibition studies are performed in conditions where TtEst2 and OPNA are at the same concentration range, i.e., under second order conditions. In these conditions, it is possible to determine if one enantiomer or both enantiomers of OPNA are consumed. For example if one equivalent of a racemic OPNA is mixed with one equivalent of enzyme, then both enantiomers are consumed if the inhibition reaches 100%, and only one if the inhibition reaches 50%. If both enantiomers react with the enzyme at a similar rate, then the bimolecular rate constants value (k_i_) reflects the value of the racemic solution. It the rates of both enantiomers are significantly different, then the curve will potentially be biphasic, and the *k_i_* for each isomer can be determined. k_i_ were determined in these conditions for paraoxon, tabun, sarin, and cyclosarin (Figure S1; Table 1).

No enantioselective inhibition was observed, except for tabun, with a 30-fold difference in inhibition rate constants. k_i_ are one to two orders of magnitude lower than those of the physiological target, AChE. Yet, the inhibition rate is sufficiently high to allow complete scavenging of µM amounts of these OPNA in less than 10 min when the enzyme is in slight excess.

By contrast to G-agents or paraoxon, the irreversible inhibition by VX appeared to be very slow in the initial assays. At 10 µM of TtEst2, the concentration of VX had to be raised to the 100-µM range in order to observed significant inhibition (Figure S2). Thus, k_i_ could be determined under pseudo-first order conditions at a fixed timepoint (10 min incubation) with various concentrations of VX (Figure S2). VX is by far the slowest of all inhibitors tested, with k_i_ = 540 ± 15 M^−1^·min^−1^, and hence 200,000-fold slower than hAChE inhibition (Table 1).

### 2.3. Biophysical Characterization

In order to determine the oligomerization state of TtEst2 in solution, we ran size exclusion chromatography, coupled to multi-angle light scattering (SEC-MALS) analysis. The late retention time on the Superdex 75 column and the molecular weight of 36,070 ± 1875 Da resulting from the MALS are in accordance with the isolation of TtEst2 as a monomer in solution. The measured molecular weight was 14% higher than the one theoretically calculated from the primary sequence (31,570 Da), and the solution was notably polydisperse, with a $\frac{\mathrm{Mw}}{\mathrm{Mn}}$ value of 1.024.

*T. terrifontis* being a thermophilic organism, its proteins are reputed as thermostable. We screened buffer conditions to determine which one yielded the highest melting temperature (Tm) for TtEst2, i.e., a buffer condition in which the enzyme is the most thermostable. Hence, we used a thermal shift assay-based screen, with buffers ranging from pH 4.0 to pH 9.0, and different NaCl concentrations (See Table S1 for detailed screen composition). TtEst2 is highly stable, with a Tm around 82 °C in most of the conditions, and up to 85.9 °C in 50 mM HEPES pH 7.5, 1 M NaCl. Very low pHs (4.0–4.5) are not favorable, with Tm as low as 60 °C. At physiological pH (7.0–7.5), TtEst2 thermal stability remains high (>82 °C), and almost no dependence on the nature of the buffer is observed (MOPS, HEPES, Tris-HCl). Finally, increasing NaCl concentration does not significantly improve the protein stability.

### 2.4. Crystallization of TtEst2

We initially attempted to crystallize TtEst2 in the conditions reported by Sayer et al. [19], i.e., 50 mM HEPES, and 15% PEG 600, yet without success. Inspecting the ESTHER database for structures of α/β-hydrolases, at the time of the redaction of this manuscript, revealed that polyethylene glycol (PEG) was the precipitating agent for 1976 out of 2310 occurrences. Thus, we kept the 50 mM HEPES buffer condition described by Sayer et al. [19] and screened multiple PEG conditions (PEG 600, PEG 3350, PEG 6000, PEG 8000, PEG 10,000, and PEG 20,000). We did not obtain any crystal in PEG 600 (15–30%) nor PEG 6000 (10–25%), and conditions with PEG 8000 (5–20%), PEG 10,000 (5–20%), and PEG 20,000 (5–20%) systematically led to protein precipitation. However, when screening PEG 3350 (15–30%), we obtained long orthorhombic crystals at 25% PEG 3350. Based on the thermal shift assay study, we screened additional buffer conditions using the 25% PEG 3350 as precipitating agent. Screening 50 mM of MOPS pH 7.0, Tris-HCl pH 7.5, Bicine pH 8.0, and Bicine pH 8.5 we observed crystals in MOPS and both Bicine conditions. However, nucleation was less controlled than in HEPES, with multiple and smaller crystals. We thus kept the 50 mM HEPES pH 7.5, 25% PEG 3350 condition for the X-ray study of TtEst2 in complex with OPNA.

### 2.5. X-ray Structures of TtEst2 and OPNA Conjugates

#### 2.5.1. Structure of Apo-TtEst2

**Figure 4:**
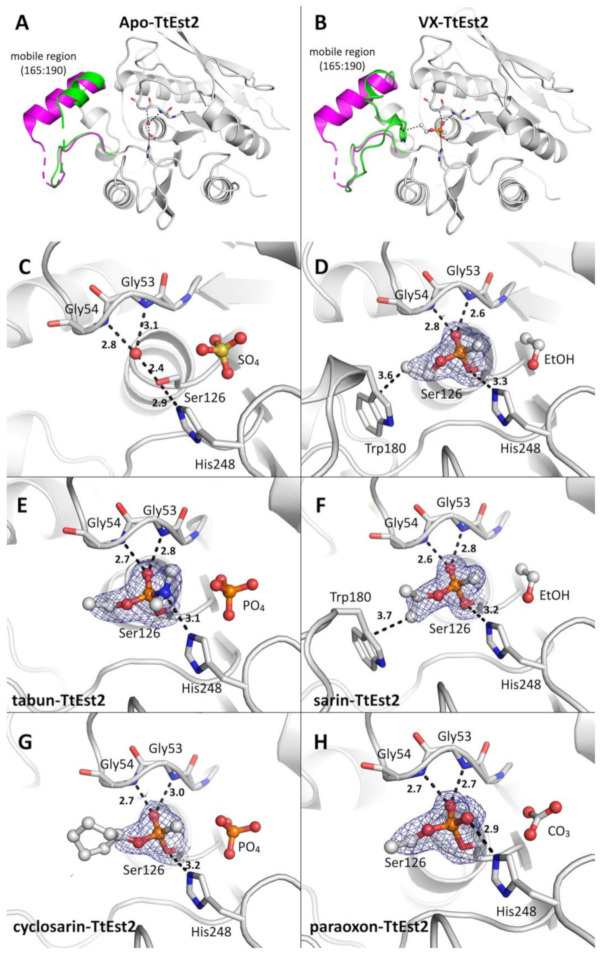
X-ray structures of apo-TtEst2 and its OPNA inhibited forms. (**A**), Overall structure of TtEst2 in the apo form. The mobile region 165–190 of the previously published structure (pdb 5ao9) is in magenta, and in green for the present work (pdb 7bfn). (**B**), Overall structure of TtEst2 phosphonylated by VX (7bfo). Mobile region 165–190 is highlighted as precedingly. (**C**–**H**), Close-up views of the TtEst2 catalytic site, with Ser126 and His248 represented as sticks, as well as Gly53 and Gly54 forming the oxyanion hole. **C**, apo-TtEst2 (7bfn); (**D**), VX-phosphonylated TtEst2 (7bfo); (**E**) tabun-phosphoramylated TtEst2 (7bft); (**F**), sarin-phosphonylated TtEst2 (7bfu); (**G**), cyclosarin-phosphonylated TtEst2 (7bfv); (**H**) Paraoxon phosphorylated TtEst2 (7bfr). The OPNA and ligands are represented in ball and stick. Nitrogen atoms are in blue, oxygen atoms in red, carbon atoms in grey, sulfur atoms in yellow, and phosphorous atoms in orange. The water molecule embedded in the oxyanion hole in the apo-TtEst is represented as a red sphere. The 2Fo-Fc electron density maps contoured at 1 σ are represented by a blue mesh for OPNA.

The orthorhombic crystals of TtEst2 of the uninhibited or apo state were obtained in conditions different that the ones previously reported by Sayer et al., where PEG 600 was used as a precipitating agent under microbatch conditions. The X-ray structure of apo TtEst2 was solved at 1.7 Å by molecular replacement using the model reported by Sayer et al., (pdb 5oa9, 1.58 Å). Data collection and refinement statistics for apo TtEst2 and the OPNA conjugates are reported in Table 2. Herein, the reported apo structure is similar to 5ao9 with a whole atom RMSD of 0.234 Å (Figure 4A).

A major difference can be observed compared to the model reported by Sayer et al. with the region comprising residues 172 to 181 that could not be modeled due to the absence of electron density. Helix 177–188 of 5ao9 appears largely unfolded in the present work, with its axis is rotated by 30°. The region 165–190 is mostly disordered with high B-factors.

Tetrahedral-shaped electron density close to the active site was observed in a feature-enhanced map [21], and modeled as a sulfate molecule (Figure 4C). Density at this location was already observed in 5ao9, and was modeled as a HEPES molecule. However, in our apo structure, there was clearly no density allowing modeling of a whole HEPES molecule. TtEst2 reportedly having no cap-domain, the active site is largely accessible to the solvent. The residues forming the catalytic triad are in an optimal configuration, with a 2.9 Å H-bond between Ser126 and His248, and a 2.9 Å H-bond between His248 and Asp216 (not shown). A water molecule sits at optimal H-bond distance between the catalytic serine, Ser126, and the main chain amide nitrogen atoms of residues Gly53 and Gly54 which form the oxyanion hole (Figure 4C).

#### 2.5.2. Structures of VX- and Sarin-TtEst2 Conjugates

Data were collected from crystals of apo-TtEst2 soaked overnight in mother liquor containing 0.5 mM VX or sarin. Structures were obtained by molecular replacement, using the apo-TtEst2 structure (pdb 7bfn) and refined to 2.0 Å and 1.65 Å for VX and sarin, respectively (Table 2). By contrast to the apo form, the mobile region 165–190 of the conjugates was sufficiently ordered to be modeled (Figure 4B, VX-TtEst2). This conformation brings Trp180 remarkably close to the active site. It is likely that this conformation is stabilized by the presence of the adduct, especially in the case of VX-TtEst2, where the region is notably much better ordered than in sarin-TtEst2.

For each structure a strong electron density was observed in the Fo–Fc map, consistent with the presence of each respective phosphyl adduct covalently linked to Ser126 (Figure 4D,F). The phosphonyl oxygen was modeled in the oxyanion hole for its stabilization through H-bonds with nitrogen amides, thus chasing the water molecule observed in the apo state. The methyl substituent of VX and sarin points toward the catalytic histidine, while the respective alkoxy substituents, ethoxy and isopropyloxy, point at the distal position of His248, toward Trp180 (3.6 and 3.7 Å). This conformation of the substituents implies the reaction with the isomers *R*_P_-VX and *R*_P_-sarin, according to a classical in-line nucleophilic substitution, with formation of a trigonal bipyramidal intermediate with both the leaving group and the catalytic serine on opposite summits. Thus, the crystalline form of the enzyme appears enantioselective for *R*_P_-VX and *R*_P_-sarin, whereas the enzyme in solution reacts at a comparable rate with *S*_P_-sarin *R*_P_-sarin according to the inhibition data. An additional peak of electron density was present in the vicinity of the adducts at the same location as the sulfate molecule in the apo form, and was modeled as an ethanol molecule according to its modest size and V-shape.

#### 2.5.3. Structure of Tabun-TtEst2, Cyclosarin, and Paraoxon Conjugates

Continuing this characterization, we solved the structures of the tabun, cyclosarin, and paraoxon conjugates. We followed a similar strategy, with overnight soaking of apo-crystals with 0.5 mM tabun or cyclosarin in mother liquor. Determination of the phases was realized by molecular replacement using the apo-model (pdb 7bfn) and the structures of tabun-, cyclosarin-, and paraoxon-TtEst2 were refined to 2.0, 1.84, and 2.0 Å, respectively (Table 2). For each of these structures, the mobile region 165–190 could not be reasonably modeled, despite the presence of multiple disconnected blobs in the Fo–Fc map, notably a peak of >4 *σ* at the location of the indole ring of Trp180 in the VX-TtEst2 structure. For tabun, the strong electron density in the Fo–Fc map allowed modeling the complete adduct (Figure 4F). Similar to VX and sarin adducts, the ethoxy substituent is a distal position of His248, while its dimethylamino points toward its. This conformation is compatible with phosphonylation by *R*_P_-Tabun. For cyclosarin, a strong electron density was observed in the Fo–Fc map, close to the catalytic serine but corresponding to the methylphosphonyl part of the adduct (Figure 4G). Modeling of the cyclohexanoxyl substituent was possible without negative peaks in the Fo–Fc maps by lowering the occupancy to 0.5. This partial occupancy is mostly likely due to disorder in absence of stabilizing interactions. Once again, the alkoxy substituent is distal to His248, while the methyl is proximal. This conformation implies that the observed conjugates are the result of phosphonylation by *R*_P_-cyclosarin. For paraoxon, the strong electron density peak at covalent distance of Ser126 was modeled as an ethylphosphoryl adduct (Figure 4H). This is consistent with the loss of the ethyl group close to the His248 through the dealkylation “aging” mechanism of cholinesterases [22]. As in all the previous structures, the additional peak of electron density in the vicinity of the three different adducts was modeled as small molecules, a phosphate with partial occupancy (0.7 and 0.75) for tabun-TtEst2 and cyclosarin-TtEst2, and a carbonate for paraoxon-TtEst2, depending on the respective shape (trigonal or tetrahedral) in the feature-enhanced maps.

## 3. Discussion and Conclusions

Using the ESTHER database [18], we searched for structurally determined esterases. We focused on thermophilic enzymes, as they allow easier and faster purification, besides displaying higher stability; similarly to what is observed for phosphotriesterases [23], bacterial enzymes known to hydrolyze OPNA. Finally, we selected an accessible active site in order to ease protein design for further optimizations. We found Esterase 2 from *T. terrifontis*, a microorganism isolated from a terrestrial hot spring in Russia [24]. Our first attempt to purify TtEst2 by heating of the crude extract was not conclusive, due to the lower thermostability compared to its homolog TtEst [25]. We switched to a conventional chromatography purification using metal chelate affinity resin, and we isolated the pure enzyme in two straightforward steps. This protocol did not copurify the 20 kDa contaminant protein observed in the heating protocol. This contaminant could be a N-terminal degradation product, or more likely a low molecular weight *E. coli* heat-shock chaperone protein. The yield was more than 60 mg per liter of autoinduction medium, however the TEV digestion of the poly-histidine tag was not complete, as shown by two bands on SDS-PAGE, and the retrieval of only 44% of the initial activity after the second chromatography step. This assumes that at least 100 mg of pure enzyme could be obtained from a single liter of medium, with better optimized conditions. Thus, despite the inability to purify the protein by heat only, this production and purification protocol are simple enough to ensure isolation of a bioscavenger at affordable cost.

Biophysical characterizations demonstrated that TtEst2 is a monomer in solution, with some polydispersity and good thermostability at physiological pH. This latter parameter could be an advantage for its use in hot locations or conditions, and ensure a good shelf life as a bioscavenger.

Enzymatic characterization showed that TtEst2 is not very efficient for substrate hydrolysis, such as pNP-C3 with a k_cat_ of about 34 s^−1^, far from the efficiency of the OPNA physiological target acetylcholinesterase for its own substrate (k_cat_ around 10^4^ s^−1^) [26]. We determined the bimolecular inhibition rate constants for five representative OPNA, namely tabun, sarin, cyclosarin, VX, and paraoxon, to estimate if this enzyme could scavenge them at a fast pace. While the reactivity was excellent for G-agent, with rate constants larger than 10^6^ M^−1^·min^−1^, and reasonable for paraoxon, with a rate constant larger than 10^4^ M^−1^·min^−1^, it was disappointingly low for VX (540 ± 15 M^−1^·min^−1^), which disqualified it in the current form from skin decontamination after exposure to this persistent agent. The lower rates for paraoxon, and specifically VX, could be related to the larger size of their leaving groups, compared to the fluoride leaving group of G-agents. Indeed, the proper orientation for in line nucleophilic substitution through a bipyramidal intermediate with the leaving group and the catalytic serine hydroxyl on opposite summits, necessitates stabilization of the larger leaving groups by interaction with residues of the enzyme. However, the active site of TtEst2 being largely open to the solvent, there are no residues that could stabilize the leaving groups of paraoxon and VX, which are in consequence freely moving in the solvent. On the contrary, these large leaving groups likely interact with residues at the enzyme surface, which leads to stabilized binding in a conformation not suitable for in line nucleophilic substitution. By contrast, the catalytic serine of hAChE is located at the bottom of a deep gorge, and OPNA like VX with large leaving groups are well stabilized by residues of the gorge, notably those of the peripheral aromatic site at the rim of the gorge [27]. This structural difference could explain the large difference in bimolecular rates observed for VX and paraoxon between hAChE and TtEst2.

Determination of the X-ray structure of apo-TtEst2 presented a structure similar to the one previously determined (pdb 5ao9), except for the mobile 165–190 region, where absence of electron density did not allow for model construction. This denotes a more mobile region, which is also described by the higher B-factors in the structure reported by Sayer et al. [19]. Thus, these residues certainly explore a wider spatial range, and such dynamic disorder could explain the polydispersity of the solution measured by SEC-MALS, by mimicking multiple species. Stabilization of the region was achieved in the presence of some adducts (VX and sarin adduct), and we suggest that mutations able to favor the conformation seen in these conjugates could enhance the scavenging of these agents.

For OPNA-inhibited forms, assuming the reaction of OPNA with TtEst2 through a S_N_2 mechanism, the configuration of the adduct implies that the enantiomers R_P_ of VX, sarin, cyclosarin, and tabun reacted with crystalline TtEst2. It is noteworthy that this geometry is not compatible with the aging mechanism, which relies on the vicinity of the catalytic histidine to assist dealkylation of alkoxy substituents [22]. By comparison, AChE denotes a strong stereoselectivity toward the S_P_ enantiomers of these OPNA [28]. It could be concluded that crystalline TtEst2 reacts with the less toxic enantiomers, which is a fatal drawback for the goal of using it for OPNA scavenging. While the inhibition study in second order conditions supports a stereoselectivity in the case of tabun, for the R_P_ enantiomer according to X-ray, it also proves an absence of stereoselectivity for sarin and cyclosarin. The question remains open for VX given that pseudo-first order conditions do not allow determining stereoselectivity. Such stereoselectivity for tabun or lack thereof for sarin and cyclosarin denotes that TtEst2 must be engineered to provide the optimal enantioselectivity, such as previously performed for OPH [15]. On the other hand, the ease of large quantity production and thermal resistance could allow the use of such an enzyme as a gentle OPNA decontaminant in conditions where current harsh solutions are not compatible, such as high molarity sodium hydroxide.

## 4. Materials and Methods

### 4.1. Recombinant Expression and Purification of TtEst2

A synthetic gene coding of TtEst2, devoid of its signal peptide and in frame with a hexa-histidine tag and tobacco etch virus (TEV) endopeptidase cleaving site at position N-terminal was purchased (GeneArt, Thermofisher Scientific, Courtaboeuf, France), and cloned between the NdeI and XhoI (New England Biolabs, Evry, France) restriction sites of the pET-17b expression vector (Novagen, Sigma-Aldrich, St Quentin Fallavier, France). After transformation into DH5α for amplification, then sequencing, the resulting vector pET-TtEst2 was transformed into *E. coli* BL21 (DE3) strain (New England Biolabs, Evry, France) for protein expression. A single clone was grown overnight in Luria broth medium containing 100 µg·mL^−1^ carbenicillin (Sigma-Aldrich, St Quentin Fallavier, France) at 37 °C. This preculture was inoculated to 1 L of autoinduction MagicMedia^TM^ *E. coli* expression medium (Thermofisher Scientific, Courtaboeuf, France) supplemented with 100 µg·mL^−1^ of carbenicillin in baffled flasks, and grown overnight at 37 °C. The bacterial pellet obtained after centrifugation was suspended into 50 mL of 20 mM HEPES pH 7.5, 150 mM NaCl (Buffer 1), and cells were lysed by sonication on ice (3 times 5 min with 0.5 s pulses and a 12.5 mm probe equipped Sonic Ruptor 400). Total soluble extracts were obtained after centrifugation (30,000× *g* at 4 °C for 30 min) and loaded onto 10 mL of cOmplete^TM^ His-Tag resin (Sigma-Aldrich, St Quentin Fallavier, France) packed in a XK 16/20 column (GE Healthcare, Velizy-Villacoublay, France) and equilibrated with Buffer 1. After extensive washing with 100 mL of Buffer 1 supplemented with 15 mM Imidazole. The enzyme was eluted with Buffer 1 supplemented with 300 mM Imidazole, and 3 mL fractions were collected. The fractions containing esterase activity were subsequently pooled. About 200 µg of recombinant poly-His-tagged TEV protease was added to the pool, which was dialyzed overnight (Slide-A-Lyzer cassette 10 kDa MWCO, Thermofisher Scientific, Courtaboeuf, France) against 2 × 2 L of Buffer 1. The solution was then loaded on the same 10 mL cOmplete^TM^ column, equilibrated, then washed, both with Buffer 1. The flow-through fraction, containing esterase activity was collected and concentrated by ultrafiltration (Amicon Ultra-15 30 kDa MWCO, Merck Millipore, Molsheim, France). Protein concentration was spectrophotometrically determined, using an absorbance at 280 nm and a calculated extinction coefficient of 1.084 (mg/mL)^−1^·cm^−1^. Aliquots of recombinant TtEst2 were flash frozen in liquid nitrogen before storage at −80 °C.

### 4.2. Acrylamide Gel Electrophoresis under Denaturing Condition (SDS-PAGE)

To assess purification, aliquots of TtEst2 were assayed by standard SDS-PAGE using precast gel (Any kD Mini-PROTEAN TGX Stain-Free, Biorad, Marnes-la-Coquette, France) after 5 min denaturation at 95 °C in denaturing and reducing conditions. After 30 min migration at 200 V the gel was revealed by UV-induced fluorescence of the tryptophan reactant included in the gel and the Gel Doc EZ imaging system (Biorad, Marnes-la-Coquette, France).

### 4.3. Enzymatic Assay and Inhibition Measurements

Esterase activity was assayed spectrophotometrically at 410 nm on a Cary 50 (Agilent, Les Ulis, France) or a Uvikon-941 (Kontron) by measuring the degradation of 1 mM of para-nitrophenyl propionate (pNP-C3; Sigma-Aldrich, St Quentin Fallavier, France) as substrate in 1 mL of Buffer 1 at 25 °C. Stock solutions of pNP-C3 were prepared in isopropanol. For Km determination, the enzymatic activity was measured over an escalating substrate concentration, ranging from 5 µM to 3 mM, and data were fit to a classical Michaelis–Menten model.

Paraoxon-ethyl was from Sigma-Aldrich, St Quentin Fallavier, France. OPNA VX, tabun, sarin cyclosarin, and VX were from INSUPE (DGA MNRBC, Vert-le-Petit, France). Stock solutions were in isopropanol.

Phosphylation rates for paraoxon, sarin, cyclosarin, and tabun were determined by incubating TtEst2 (2 or 3.9 µM) with different concentrations of OPNA (from 0.5 µM to 8 µM, depending on the inhibitor) in 500 µL of Buffer 1, and measuring the esterase residual activity of 50-µL aliquots at various times after initiation of the inhibition reaction. In these conditions, there is not a large excess of inhibitor, and the concentrations of both the enzyme and the inhibitor vary significantly during the time course of the inhibition. Therefore, the normalized residual activity as a function of time follows second order kinetics, described by the following equation:

(1) $$\frac{\left[E\right]}{{\left[E\right]}_{0}}=\frac{\left({\left[I\right]}_{0}-{\left[E\right]}_{0}\right)e^{-k_{i}\left({\left[I\right]}_{0}-{\left[E\right]}_{0}\right)t}}{{\left[I\right]}_{0}-{\left[E\right]}_{0}e^{-k_{i}\left({\left[I\right]}_{0}-{\left[E\right]}_{0}\right)t}}$$

where [E] is the concentration of residual active enzyme at time = t, [E]_0_ the initial concentration of enzyme, [I]_0_ the initial concentration of racemic inhibitor, and k_i_ the bimolecular inhibition rate constant.

For paraxon, sarin, and cyclosarin, k_i_ was determined using the equation above and the solver of Excel, with all the measured residual activities for every inhibitor concentrations tested.

For tabun, the curves were biphasic due to a higher phosphylation rate for one of the two tabun enantiomers. In these conditions, *k_i_*_1_ and *k_i_*_2_ for each enantiomers were determined by fitting the experimental data against numerical solutions of the set of differential equations describing the kinetic system using Pro fit (Quantumsoft):

(2) $$\frac{d\left[E\right]}{\mathrm{dt}}=-k_{i1}\left[E\right]\left[I_{1}\right]-k_{i2}\left[E\right]\left[I_{2}\right]$$

(3) $$\frac{d\left[I_{1}\right]}{\mathrm{dt}}=-k_{i1}\left[E\right]\left[I_{1}\right]$$

(4) $$\frac{d\left[I_{2}\right]}{\mathrm{dt}}=-k_{i2}\left[E\right]\left[I_{2}\right]$$

where [E], [I_1_], and [I_2_] are, respectively, the concentration of residual active enzyme, enantiomer 1 and enantiomer 2 at time = t, and k_i1_ and k_i2_ the bimolecular inhibition rate constants of the enantiomers.

For VX, as the progressive inhibition was very slow, k_i_ could be determined under pseudo-first order conditions. About 10 µM of TtEst2 was incubated with VX concentrations ranging from 100 to 400 µM for 10 min, and 10 µL aliquots were used to measure esterase activity in 1 mL (100-fold dilution). The pseudo-first order inhibition can be described by the following classical equation:

(5) $$\frac{\left[E\right]}{{\left[E\right]}_{0}}=e^{-k_{i}\left[\mathrm{VX}\right]t}$$

where [E] is the concentration of residual active enzyme at time t = 10 min, [E]_0_ the initial concentration of enzyme, k_i_ the bimolecular inhibition rate constant, and t = 10 min, i.e., the time at which the reaction is stopped by dilution for the measurement of activity. Normalized residual activity at t = 10 min in function of [VX] was fitted by non-linear regression to determine k_i_ using Pro fit (Quantumsoft).

### 4.4. Size Exclusion Chromatography Coupled to Multi-Angle Light Scattering (SEC-MALS)

For determination of the oligomeric state in solution, 50 µL of pure TtEst2 at 2 mg·mL^−1^ was injected into a Superdex 75 Increase 10/300 GL (GE Healthcare, Velizy-Villacoublay, France) developed in 20 mM HEPES pH 7.5, 150 mM NaCl at a flow rate of 0.5 mL.min^−1^ on an AKTA purified (GE Healthcare, Velizy-Villacoublay, France) multi-angle laser light scattering was measured in-line using a 8 angles DAWN^®^ Heleos^®^ II detector (Wyatt Technologies Corp., Toulouse, France) and a 663 nm laser light, and coupled to a Optilab^®^ T-rEx differential refractometer (Wyatt Technologies Corp., Toulouse, France) for refractive index measurements. Data were analyzed with Astra^®^ v6.1 software (Wyatt Technologies Corp., Toulouse, France) using a refractive index increment ($\frac{dn}{dc}$) of 0.190 mL·g^−1^, a mean value usually accepted for proteins [29].

### 4.5. Thermal Shift Assay

To study TtEst2 thermal stability, a thermal shift assay (TSA, or differential scanning fluorimetry) was realized on a StepOne Plus Real Time PCR system (Thermofischer Scientific, Courtaboeuf, France) using a home-made buffer screen inspired by one reported by Boivin et al. [30], and similarly to what has been previously technically reported [31].

### 4.6. Protein Crystallization and Crystal Inhibitions

Crystals of TtEst2 were obtained by hanging-drop vapor diffusion by mixing an equal volume of a 0.1 µm filtered protein solution concentrated with 20 mg·mL^−1^ and 50 mM HEPES pH 7.5, 25% PEG 3350 solution as crystallization buffer, and growing at 20 °C. Crystal inhibition was realized by soaking using a 0.5 mM OPNA solution in crystallization buffer. Crystals were cryoprotected using Paratone^®^ N (Hampton Research, Aliso Viejo, CA, USA) before flash cooling into liquid nitrogen.

### 4.7. Structure Determination

X-ray diffraction data were recorded at 100 K on the Proxima-1 or Proxima-2A beamlines of the SOLEIL Synchrotron (Saint Aubin, France), at a wavelength close to 0.98 Å. Images recorded on an EIGER detector were processed using XDS [32], manually applying CC1/2 [33] and I/sigma > 1 as cutoff or automatically using XDSME [34]. Data were further processed using the Phenix software suite [35]. Phases were determined by molecular replacement using the TtEst2 structure (PDB entry 5ao9) devoid of water molecules and ligands. The models were constructed by iterative cycles of refinement (Phenix.refine), and model building using Coot software [36]. Phosphyl-serine adduct restraints were generated for each OPNA using Phenix.eLBOW [37] and the AM1 semi-empirical quantum mechanical method. Structures of apo TtEst2 and its conjugates with VX, tabun, sarin, cyclosarin, and paraoxon were deposited in the Protein Data Bank under accession numbers 7bfn, 7bfo, 7bft, 7bfu, 7bfv, and 7bfr respectively. Structure representations were made with PyMOL (Schrodinger LLC).

## Figures and Tables

**Table 1:** Bimolecular rates of inhibition (k_i_) of TtEst2 for various OPNA.

	k_i_ (M^−1^·min^−1^)		
OPNA	TtEst2	hAChE from [20]	hAChE/TtEst2
POX	2.41 ± 0.12 × 10^4^	2.2 × 10^6^	91
GA	1.6 ± 0.4 × 10^6^/5.1 ± 0.8 × 10^4^	7.4 × 10^6^	5/145
GB	1.51 ± 0.12 × 10^6^	2.7 × 10^7^	18
GF	1.29 ± 0.11 × 10^6^	4.9 × 10^8^	230
VX	540 ± 15	1.2 × 10^8^	220,000

**Table 2:** Data collection and refinement statistics calculated using Phenix. R-work = Σ |Fo|−|Fc|/Σ |Fo|, where Fo and Fc are observed and calculated structure factors. R-free set uses about 1000 randomly chosen reflections. Statistics for the highest-resolution shell are shown in parentheses.

Ligand	Apo	VX	Tabun	Sarin	Cyclosarin	Paraoxon
Pdb Code	7bfn	7bfo	7bft	7bfu	7bfv	7bfr
**Data Collection**						
wavelength (Å)	0.9786	0.9801	0.9801	0.9789	0.9786	0.9801
Resolution range (Å) (highest-resolution shell)	33.85–1.7 (1.77–1.7)	42.11–1.994 (2.065–1.994)	37.47–1.993 (2.064–1.993)	36.5–1.65 (1.709–1.65)	33.87–1.84 (1.906–1.84)	36.74–1.99 (2.061–1.99)
space group, mol/AU	P 21 21 21	P 21 21 21	P 21 21 21	P 21 21 21	P 21 21 21	P 21 21 21
unit cell parameters (Å)	56.21 67.7 74.58 90 90 90	53.7 67.84 75.76 90 90 90	53.74 67.87 74.94 90 90 90	52.86 67.71 75.71 90 90 90	55.83 67.74 74.52 90 90 90	53.52 67.64 75.97 90 90 90
Total reflections	405,998 (28,562)	253,233 (23,090)	247,497 (21,226)	375,032 (31,442)	325,398 (31,661)	249,281 (19,651)
Unique reflections	31,810 (2990)	19,424 (1872)	19,260 (1851)	32,804 (3175)	25,095 (2435)	19,251 (1664)
Multiplicity	12.8 (9.5)	13.0 (12.3)	12.9 (11.5)	11.4 (9.8)	13.0 (13.0)	12.9 (11.8)
Completeness (%)	98.66 (94.94)	99.75 (98.37)	99.54 (96.90)	98.07 (96.59)	99.26 (97.41)	98.59 (86.79)
Mean I/σ (I)	13.71 (1.07)	29.15 (9.54)	17.14 (5.33)	17.40 (1.14)	22.16 (4.76)	27.16 (8.19)
Wilson B-factor	35.45	26.79	25.37	26.44	33.92	25.44
R-merge	0.0860 (1.603)	0.0597 (0.2776)	0.1300 (0.5887)	0.08227 (1.503)	0.06329 (0.397)	0.0801 (0.3037)
R-meas	0.0897 (1.694)	0.0622 (0.2896)	0.1356 (0.6157)	0.08608 (1.584)	0.06611 (0.414)	0.0835 (0.3169)
R-pim	0.0251 (0.5321)	0.0174 (0.0810)	0.0380 (0.1758)	0.0248 (0.4871)	0.01879 (0.115)	0.0232 (0.0890)
CC1/2	0.998 (0.776)	0.999 (0.99)	0.998 (0.978)	0.999 (0.641)	0.999 (0.99)	0.999 (0.991)
CC *	0.999 (0.935)	1 (0.997)	0.999 (0.995)	1 (0.884)	1 (0.998)	1 (0.998)
**Refinement Statistics**						
Reflections used	31,554 (2986)	19,410 (1871)	19,232 (1844)	32,727 (3175)	24,979 (2408)	19,232 (1662)
Reflections for R-free	1264 (119)	972 (94)	962 (93)	1310 (127)	1249 (122)	961 (83)
R-work	0.2316 (0.4505)	0.1601 (0.1708)	0.1595 (0.1859)	0.1785 (0.4862)	0.1802 (0.2521)	0.1611 (0.1760)
R-free	0.2622 (0.4827)	0.2034 (0.2237)	0.1928 (0.2294)	0.2204 (0.4904)	0.2273 (0.2804)	0.2073 (0.2858)
CC(work)	0.954 (0.783)	0.970 (0.956)	0.966 (0.953)	0.970 (0.442)	0.962 (0.960)	0.965 (0.962)
CC(free)	0.954 (0.780)	0.849 (0.827)	0.966 (0.920)	0.978 (0.269)	0.925 (0.914)	0.968 (0.844)
Number of non-H atoms	2187	2435	2315	2461	2266	2261
macromolecule	2092	2191	2079	2187	2064	2037
ligands	5	3	5	3	6	10
solvent	90	241	231	271	196	214
Protein residues	268	277	264	276	264	260
RMSD (bonds; Å)	0.008	0.004	0.008	0.008	0.011	0.009
RMSD (angles; deg)	1.02	0.75	1.06	1.02	1.13	1.05
Ramachandran favored (%)	94.70	96.69	97.67	96.31	96.89	96.88
Ramachandran allowed (%)	5.30	2.57	2.33	2.95	3.11	3.12
Ramachandran outliers (%)	0.00	0.74	0.00	0.74	0.00	0.00
Rotamer outliers (%)	0.00	0.45	0.47	2.25	0.48	0.48
Clashscore	7.19	6.17	6.26	7.79	5.11	4.67
Average B-factor (Å^2^)	64.89	36.62	32.09	35.57	52.22	30.33
macromolecules (Å^2^)	65.30	36.14	31.27	34.95	52.18	29.53
ligands (Å^2^)	64.92	25.64	28.22	26.64	47.00	29.53
solvent (Å^2^)	55.28	41.16	39.61	40.67	52.82	37.91
Number of TLS groups	1	1	1	1	1	1

## Data Availability

Structure data are available in the Protein Data Bank (https://www.rcsb.org).

## Supplementary Materials

The following are available online at, Figure S1: Progressive inhibition of TtEst2 by paraoxon, sarin, cyclosarin and tabun. Figure S2: Inhibition of TtEst2 by VX. Table S1: Composition of the 96-well format buffer screen.

## Sample Availability

Samples of the compounds are not available from the authors.
