# Supplementary material for: A Thermophilic Bacterial Esterase for Scavenging Nerve Agents: A Kinetic, Biophysical and Structural Study
Source: Molecules. 2021 Jan 27;26(3):657. doi: 10.3390/molecules26030657 (PMC7865465; doi:10.3390/molecules26030657)
Supplement: Supplementary file 1 [file molecules-26-00657-s001.pdf]

Article

A Thermophilic Bacterial Esterase for Scavenging Nerve Agents: A Kinetic, Biophysical and Structural Study

Janek Bzdrenga, Elodie Trenet, Fabien Chantegreil, Kevin Bernal, Florian Nachon * and Xavier Brazzolotto

Département de Toxicologie et Risques Chimiques, Institut de Recherche Biomédicale des Armées, Brétigny-sur-Orge 91220, France; janek.bzdrenga@def.gouv.fr (J.B.); elodie.trenet@chemdef.fr (E.T.); fabien.chantegreil@def.gouv.fr (F.C.); kevin.bernal@chemdef.fr (K.B.); xavier.brazzolotto@def.gouv.fr (X.B.)
* Correspondence: florian.nachon@def.gouv.fr

Supplementary Materials

- Figure S1: Progressive inhibition of TtEst2 upon incubation with various concentrations of paraoxon, sarin, cyclosarin and tabun.
- Figure S2. Inhibition of TtEst2 by VX.
- Table S1. Composition of the 96-well format buffer screen used for the TSA study.

Figure S1

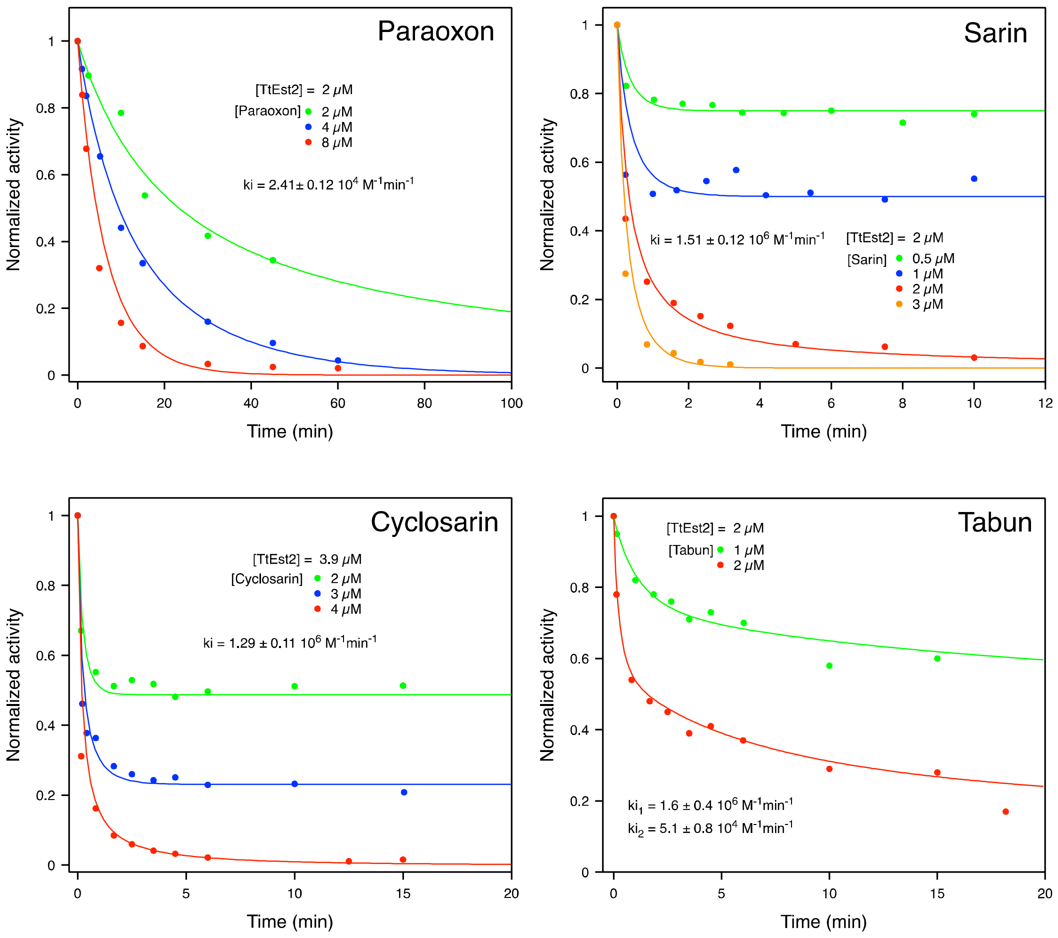

Figure S1. Progressive inhibition of TtEst2 upon incubation with various concentrations of paraoxon, sarin, cyclosarin and tabun. Phosphylation rates for paraoxon, sarin, cyclosarin and tabun were determined by incubating TtEst2 (2 or 3.9 µM) with different concentrations of agent of the same order, and measuring the esterase residual activity of aliquots at various times after initiation of inhibition reaction. In these second order conditions, the normalized residual activity as a function of time is described by the following equation:

$$\frac{[E]}{[E]_0} = \frac{([I]_0 - [E]_0)e^{-k_i([I]_0-[E]_0)t}}{[I]_0 - [E]_0 e^{-k_i([I]_0-[E]_0)t}} \qquad (1)$$

Where [E] is the concentration of residual active enzyme at time=t, $[E]_0$ the initial concentration of enzyme, $[I]_0$ the initial concentration of racemic inhibitor, and k_i the bimolecular inhibition rate constant. k_i was determined for paraxon, sarin and cyclosarin, using the equation above and the solver of Excel, including all the residual activity for every concentration tested for one particular inhibitor.

For tabun, the curves are biphasic, thus indicating that the two isomer inhibit TtEst2 at a significantly different rate. In these conditions, ki1 and ki2, the bimolecular inhibition rate constants of each enantiomer were determined by fitting the experimental data against numerical solutions of the set of differential equations describing the kinetic system using Pro fit (Quantumsoft):

$$\frac{d[E]}{dt} = -k_{i1}[E][I_1] - k_{i2}[E][I_2] \qquad (2)$$

$$\frac{d[I_1]}{dt} = -k_{i1}[E][I_1] \quad (3)$$

$$\frac{d[I_2]}{dt} = -k_{i2}[E][I_2] \quad (4)$$

Where [E], [I_1] and [I_2] are respectively the concentration of residual active enzyme, enantiomer 1 and enantiomer 2 at time = t.

Figure S2

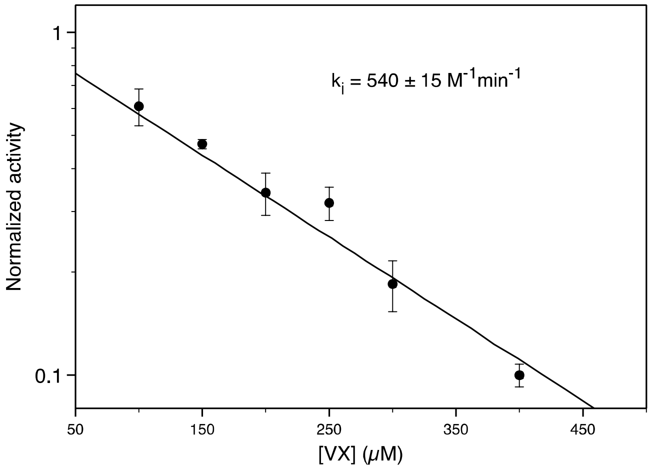

Figure S2. Inhibition of TtEst2 by VX. Residual activity of 10 μM TtEst2, at the 10-min time point of incubation with increasing concentrations of a large excess of VX was measured in triplicate using 10-μL aliquots diluted in a 1-mL cuvette. Error bars represents the standard deviation. In these conditions, the kinetic of inhibition is of the pseudo-first order type and the rate of inhibition is calculated by nonlinear regression using the following classical equation:

$$\frac{[E]}{[E]_0} = e^{-k_i[VX]t} \quad (2)$$

Where [E] is the concentration of residual active enzyme at time t = 10 min, $[E]_0$ the initial concentration of enzyme, [VX] the concentration of VX, k_i the bimolecular inhibition rate constant, and t = 10 min, i.e. the time at which the reaction is stopped by dilution for the measurement of activity. The line represents the calculation of the normalized activity using the fitted parameter k_i.

Table S1. Composition of the 96-well format buffer screen used for the TSA study.

	1	2	3	4	5	6	7	8	9	10	11	12
**A**	Water	Citrate pH 4.0	Na Acetate pH 4.5	Citrate pH 5.0	MES pH 6.0	KH2PO4 pH 6.0	Citrate pH 6.0	Bis-Tris pH 6.5	Cacodylate pH 6.5	NaH2PO4 pH 7.0	KH2PO4 pH 7.0	HEPES pH 7.0
**B**	MOPS pH 7.0	Am. Acetate pH 7.3	Tris-HCl pH 7.5	NaH2PO4 pH 7.5	HEPES pH 7.5	Imidazole pH 8.0	Tris-HCl pH 8.0	Tricine pH 8.0	Bicine pH 8.0	Bicine pH 8.5	Tris-HCl pH 8.5	Bicine pH 9.0
**C**	Water 250 mM NaCl	Citrate pH 4.0 250 mM NaCl	Na Acetate pH 4.5 250 mM NaCl	Citrate pH 5.0 250 mM NaCl	MES pH 6.0 250mM NaCl	KH2PO4 pH 6.0 250 mM NaCl	Citrate pH 6.0 250 mM NaCl	Bis-Tris pH 6.5 250 mM NaCl	Cacodylate pH 6.5 250 mM NaCl	NaH2PO4 pH 7.0 250 mM NaCl	KH2PO4 pH 7.0 250 mM NaCl	HEPES pH 7.0 250 mM NaCl
**D**	MOPS pH 7.0 250 mM NaCl	Am. Acetate pH 7.3 250 mM NaCl	Tris-HCl pH 7.5 250 mM NaCl	NaH2PO4 pH 7.5 250 mM NaCl	HEPES pH 7.5 250 mM NaCl	Imidazole pH 8.0 250 mM NaCl	Tris-HCl pH 8.0 250 mM NaCl	Tricine pH 8.0 250 mM NaCl	Bicine pH 8.0 250 mM NaCl	Bicine pH 8.5 250 mM NaCl	Tris-HCl pH 8.5 250 mM NaCl	Bicine pH 9.0 250 mM NaCl
**E**	10 mM MES pH 6.0	50 mM MES pH 6.0	100 mM MES pH 6.0	250 mM MES pH 6.0	10 mM KH2PO4 pH 6.0	50 mM KH2PO4 pH 6.0	100 mM KH2PO4 pH 6.0	250 mM KH2PO4 pH 6.0	10 mM Citrate pH 6.0	50 mM Citrate pH 6.0	100 mM Citrate pH 6.0	250 mM Citrate pH 6.0
**F**	10 mM NaH2PO4 pH 7.5	50 mM NaH2PO4 pH 7.5	100 mM NaH2PO4 pH 7.5	250 mM NaH2PO4 pH 7.5	10 mM HEPES pH 7.5	50 mM HEPES pH 7.5	100 mM HEPES pH 7.5	250 mM HEPES pH 7.5	10 mM Tris-HCl pH 7.5	50 mM Tris-HCl pH 7.5	100 mM Tris-HCl pH 7.5	250 mM Tris-HCl pH 7.5
**G**	50 mM MES pH 6.0 50 mM NaCl	50 mM MES pH 6.0 125 mM NaCl	50 mM MES pH 6.0 250 mM NaCl	50 mM MES pH 6.0 500 mM NaCl	50 mM MES pH 6.0 750 mM NaCl	50 mM MES pH 6.0 1 M NaCl	50 mM MOPS pH 7.0 50 mM NaCl	50 mM MOPS pH 7.0 125 mM NaCl	50 mM MOPS pH 7.0 250 mM NaCl	50 mM MOPS pH 7.0 500 mM NaCl	50 mM MOPS pH 7.0 750 mM NaCl	50 mM MOPS pH 7.0 1 M NaCl
**H**	50mM HEPES pH 7.5 50 mM NaCl	50mM HEPES pH 7.5 125 mM NaCl	50mM HEPES pH 7.5 250 mM NaCl	50mM HEPES pH 7.5 500 mM NaCl	50mM HEPES pH 7.5 750 mM NaCl	50mM HEPES pH 7.5 1 M NaCl	50 mM Tris-HCl pH 8.0 50 mM NaCl	50 mM Tris-HCl pH 8.0 125 mM NaCl	50 mM Tris-HCl pH 8.0 250 mM NaCl	50 mM Tris-HCl pH 8.0 500 mM NaCl	50 mM Tris-HCl pH 8.0 750 mM NaCl	50 mM Tris-HCl pH 8.0 1 M NaCl
